# PCK1 negatively regulates cell cycle progression and hepatoma cell proliferation via the AMPK/p27^Kip1^ axis

**DOI:** 10.1186/s13046-019-1029-y

**Published:** 2019-02-04

**Authors:** Lin Tuo, Jin Xiang, Xuanming Pan, Jieli Hu, Hua Tang, Li Liang, Jie Xia, Yuan Hu, Wenlu Zhang, Ailong Huang, Kai Wang, Ni Tang

**Affiliations:** Key Laboratory of Molecular Biology for Infectious Diseases (Ministry of Education), Institute for Viral Hepatitis, Department of Infectious Diseases, The Second Affiliated Hospital, Chongqing Medical University, Chongqing, People’s Republic of China

**Keywords:** Phosphoenolpyruvate carboxykinase 1, Hepatocellular carcinoma, Cell cycle arrest, CDK/Rb/E2F pathway, AMP-activated protein kinase

## Abstract

**Background:**

Altered glucose metabolism endows tumor cells with metabolic flexibility for biosynthesis requirements. Phosphoenolpyruvate carboxykinase 1 (PCK1), a key enzyme in the gluconeogenesis pathway, is downregulated in hepatocellular carcinoma (HCC) and predicts poor prognosis. Overexpression of PCK1 has been shown to suppress liver tumor growth, but the underlying mechanism remains unclear.

**Methods:**

mRNA and protein expression patterns of PCK1, AMPK, pAMPK, and the CDK/Rb/E2F pathway were determined using qRT-PCR and western blotting. Cell proliferation ability and cell cycle were assessed by MTS assay and flow cytometric analysis. The effect of PCK1 on tumor growth was examined in xenograft implantation models.

**Results:**

Both gain and loss-of-function experiments demonstrated that PCK1 deficiency promotes hepatoma cell proliferation through inactivation of AMPK, suppression of p27^Kip1^ expression, and stimulation of the CDK/Rb/E2F pathway, thereby accelerating cell cycle transition from the G1 to S phase under glucose-starved conditions. Overexpression of PCK1 reduced cellular ATP levels and enhanced AMPK phosphorylation and p27^Kip1^ expression but decreased Rb phosphorylation, leading to cell cycle arrest at G1. AMPK knockdown significantly reversed G1-phase arrest and growth inhibition of PCK1-expressing SK-Hep1 cells. In addition, the AMPK activator metformin remarkably suppressed the growth of PCK1-knockout PLC/PRF/5 cells and inhibited tumor growth in an orthotropic HCC mouse model.

**Conclusion:**

This study revealed that PCK1 negatively regulates cell cycle progression and hepatoma cell proliferation via the AMPK/p27^Kip1^ axis and supports a potential therapeutic and protective effect of metformin on HCC.

**Electronic supplementary material:**

The online version of this article (10.1186/s13046-019-1029-y) contains supplementary material, which is available to authorized users.

## Background

Metabolic reprogramming or “deregulating cellular energetics” is a hallmark of cancer [1]. Tumor cells undergo aerobic glycolysis even in the presence of oxygen, also known as the Warburg effect, which is critical for meeting the metabolic requirements of rapid cancer cell growth and proliferation [2]. The liver is the major site of gluconeogenesis during fasting, where the conversion of oxaloacetate (OAA) and GTP to phosphoenolpyruvate (PEP) and GDP by phosphoenolpyruvate carboxykinase (PCK; also known as PEPCK, EC number 4.1.1.32) is the first rate-limiting step in hepatic gluconeogenesis [3, 4]. Two isoforms of PCK have been described, a cytoplasmic form (PCK1, PEPCK-C) and a mitochondrial isoform (PCK2, PEPCK-M) [5]. In the mammalian liver, PCK1 accounts for over 95% of gluconeogenesis activity [6], is upregulated in colon and melanoma cancers, and is associated with increased glucose consumption to support anabolic metabolism and tumor growth. However, in hepatocellular carcinoma (HCC), the expression levels of PCK and other key gluconeogenic enzymes are strikingly suppressed [7, 8], indicating that PCK has contrasting roles in tumorigenesis in different organs. Nevertheless, the role of PCK in HCC progression remains incompletely understood.

Recent metabolomic studies confirmed that glucose levels in tumors are generally lower than those in non-transformed tissues [9, 10]. However, cancer cells require sufficient glucose to facilitate rapid tumor growth through the generation of building blocks, which are necessary for the synthesis of essential cellular components [9, 11]. Therefore, cancer cells require delicate energy sensing and metabolic adaptation pathways. Mammalian AMP-activated protein kinase (AMPK) is a central cellular sensor of adenine nucleotides that plays a key role in sensing cellular energy availability and regulating metabolic adaptation pathways [11, 12]. Energy stress conditions in tumor tissues are sensed by AMPK through elevated intracellular AMP/ATP ratios. Once activated, phosphorylated AMPK acts to restore the energy balance by increasing catabolic ATP-generating processes, such as glycolysis and fatty-acid oxidation, while decreasing ATP-consuming processes, such as fatty acid synthesis and cell proliferation [13]. AMPK activation has been reported to suppress cell proliferation by regulating cell cycle progression or inhibiting the synthesis of certain proteins, such as p27^Kip1^ [14–16].

In this study, we explored the potential role of PCK1 in the suppression of cellular proliferation and tumor growth in HCC. Interestingly, we found that PCK1 activates AMPK and delays G1 to S phase (G1/S) transition. Our study also revealed that the AMPK activator metformin remarkably suppresses tumor growth in an orthotropic HCC mouse model.

## Methods

### Patient samples

Liver tumor specimens and adjacent non-tumorous specimens were collected from 20 patients who underwent surgical resection at the Second Affiliated Hospital of Chongqing Medical University (Chongqing, China). Patients had not received chemotherapy or radiation therapy before surgery. The samples were frozen immediately after surgery and stored in liquid nitrogen. All patients provided informed consent and the study was conducted with the approval of the Institutional Ethical Review Board of Chongqing Medical University (project license number: 2017012).

### Cell cultures and drug treatment

The human hepatoma cell lines HepG2, SK-Hep1, and PLC/PRF/5 were obtained from the American Type Culture Collection (ATCC; Manassas, VA, USA). Other hepatoma cell lines like Huh7 and normal human liver cells MiHA were obtained from the Cell Bank of the Chinese Academy of Sciences (Shanghai, China). Apart from HepG2, cells were cultured in Dulbecco’s modified Eagle’s medium (DMEM; Hyclone, Logan, UT, USA) supplemented with 10% fetal bovine serum (FBS; Gibco, Rockville, MD, USA), 100 IU penicillin, and 100 mg/mL streptomycin. HepG2 cells were cultured in minimum essential medium (MEM; Hyclone). Cell lines involved in this study were recently authenticated by short tandem repeat (STR) profiling (Beijing Microread Gene Technology Co., Beijing, China). All cells were incubated in a humidified atmosphere at 37 °C containing 5% CO_2_.

For cell cycle analysis, cells were collected at different time points post-synchronization. For low-glucose treatment, cells were incubated in glucose-free DMEM (Hyclone) supplemented with 10% FBS, 100 IU penicillin, 100 mg/mL streptomycin, and the indicated concentrations of glucose (Hyclone) for 12 h. For immunoblotting and cell cycle analysis under glucose-deprived conditions, cells were incubated in DMEM containing 1 mM glucose and metformin (5 mM; Cat# S1950; Selleck, Houston, TX, USA) for 12 h and then collected for analysis. For proliferation and colony formation assays, cells were cultured in normal DMEM supplemented with 0.5 mM metformin.

### Plasmid constructs and adenovirus production

The full-length cDNA of PCK1 (coding sequence of NM_002591) was amplified from plasmid pOTB7-PCK1 (Cat# FL07339; GeneCopoeia, Rockville, MD, USA). Primers are listed in Additional file 1: Table S1. The amplified PCK1 fragment was inserted into the shuttle vector pAdTrack-TO4 (kindly provided by Dr. Tong-Chuan He, University of Chicago, USA). Adenoviral recombinant pAd-PCK1 was generated using the AdEasy system as described previously [17]. The analogous adenovirus expressing GFP only (AdGFP) was used as control.

### Lentivirus construction

Two pairs of oligonucleotides encoding short hairpin RNA (shRNA) targeting isoform α1 of AMPK (Additional file 1: Table S1) were designed and subcloned into the lentiviral vector pLL3.7 (kindly gifted by Prof. Bing Sun from the Shanghai Institute of Biochemistry and Cell Biology, Chinese Academy of Sciences, China) to generate shAMPK lentivirus. A control virus (shControl), with a scrambled shRNA insert, was also generated. For lentiviral supernatant production, HEK-293 T cells were transfected with 2 μg VSVG, 3 μg Δ8.9, and 4 μg of the shRNA lentiviral construct for lentiviral package using Lipofectamine 2000 reagent (Thermo Fisher Scientific, Waltham, MA, USA), according to manufacturer’s instructions. SK-Hep1 cells were transduced with the packaged lentiviruses in the presence of 5 μg/mL polybrene.

### RNA-sequencing (RNA-seq) and expression analysis

Hepatoma cells were infected with AdGFP or AdPCK1 for 36 h and then RNA was extracted using TRIzol reagent (Invitrogen, Rockville, MD, USA) according to manufacturer’s instructions. RNA-seq and bioinformatic data analysis were conducted at Shanghai Novel Bio Ltd. (Shanghai, China). Strand-specific RNA-seq libraries were prepared using the Total RNA-seq (H/M/R) Library Prep Kit (Vazyme Biotech, Nanjing, China) and were sequenced on an Ion Torrent Proton Sequencer (Life Technologies, Carlsbad, CA, USA) according to the Ion PI Sequencing 200 Kit v2.0 (Life Technologies). Raw reads in FASTQ format were quality-controlled using FastQC (http://www.bioinformatics.babraham.ac.uk/projects/fastqc/). RNA-seq reads were aligned to the reference genome using Bowtie and uniquely mapped reads were used for further analysis. Gene expression levels were expressed as reads per kilobase per million reads (RPKM) and differences in gene expression were calculated with rSeq (http://www-personal.umich.edu/~jianghui/rseq/).

### RNA extraction and real-time reverse transcription (qRT)-PCR

Total cellular RNA was extracted from cultured cells using TRIzol reagent (Invitrogen) and then reverse transcribed using Moloney murine leukemia virus reverse transcriptase (Promega, Madison, WI, USA) and random hexamers (Promega). The synthesized cDNA was then used as template for qPCR of the respective genes using SYBR Green and a CFX Real-Time PCR Detection System (Bio-Rad Laboratories, Hercules, CA, USA). The actin beta gene (*ACTB*) was used as a reference gene for normalization. Relative mRNA levels were calculated using the 2^–ΔΔCt^ method. All primers are listed in Additional file 1: Table S1.

### Western blot analysis

Protein was extracted from cells or tissue samples using lysis buffer (Beyotime, Shanghai, China) supplemented with 1 mM phenylmethylsulfonyl fluoride (Beyotime), after which the protein concentration was determined using a BCA protein assay kit (Beyotime). Proteins were resolved by sodium dodecyl sulfate polyacrylamide gel electrophoresis and electrotransferred to polyvinylidene difluoride membranes (Millipore, Billerica, MA, USA). The membranes were incubated with primary antibodies against PCK1 (1:1000; Cat# BS6870; Bioworld, Atlanta, GA, USA), phospho-AMPKα1 (T172; 1:1000; Cat# 2535; Cell Signaling Technology, Danvers, MA, USA), AMPKα (1:1000; Cat# 2795; Cell Signaling Technology), p27^Kip1^(1:1000; Cat# 3686; Cell Signaling Technology), phospho-Rb (S780; 1:1000; Cat# BS4164; Bioworld), Rb (T774; 1:1000; Cat# BS1310; Bioworld), cyclin E1 (L389;1:1000; Cat# BS1086; Bioworld), CDK2 (78B2; 1:1000; Cat# 2546; Cell Signaling Technology), phospho-CDK2 (Thr160; 1:1000; Cat# 2561; Cell Signaling Technology), E2F2 (K236; 1:1000; Cat# BS2057; Bioworld), LKB1 (1:1000; Cat# GTX130697; GeneTex, Irvine, CA, USA), and CAMKK2 (1:1000; Cat# GTX115461; GeneTex). Then, the membranes were incubated with the corresponding horseradish peroxidase-conjugated secondary antibody (Abcam, Cambridge, UK). Proteins were detected using the Super Signal West Pico Chemiluminescent Substrate Kit (Millipore) and quantified by densitometry using ImageJ software (National Institutes of Health, Bethesda, MA, USA; http://imagej.nih.gov/). GAPDH (Cat# AF0006; Beyotime) or β-actin (Cat# BL005B; Biosharp) were used as internal control. All experiments were independently repeated three times.

### Histological and immunohistochemistry (IHC) analysis

Liver samples were fixed in fresh 4% paraformaldehyde and subjected to routine histological procedures for embedding in paraffin. Then, the samples were cut in 4.5 μm-thick sections that were processed for hematoxylin and eosin (HE) or IHC staining with antibodies targeting PCK1 (1:500), pAMPK (1:200), or p27^Kip1^ (1:500). For IHC assays, the sections were incubated with secondary anti-rabbit IgG (ZSGB-BIO, Beijing, China) and stained with 3,3′-diaminobenzidine (ZSGB-BIO). Stained slides were scanned with a Pannoramic Scan 250 Flash or MIDI system and images were acquired using Pannoramic Viewer 1.15.2 (3DHistech, Budapest, Hungary).

### CRISPR/Cas9-mediated knockout of PCK1

The CRISPR/Cas9 plasmids lentiCRISPR v2, pMD2.G, and psPAX2 were kindly provided by Prof. Ding Xue from the School of Life Sciences, Tsinghua University (Beijing, China). Single-guide RNAs targeting human PCK1 were designed using the E-CRISP online tool (http://www.e-crisp.org/E-CRISP/designcrispr.html). The PCK1 targeting sequences (listed in Additional file 1: Table S1) were synthesized by TsingKe Biological Technology (Chongqing, China) and cloned into lentiCRISPR v2 vectors. Lentiviruses were generated by co-transfecting HEK293T cells with lentiCRISPR v2, envelop plasmid pMD2.G, and packaging plasmid psPAX2 using Lipofectamine 2000 according to manufacturer’s instructions. After 48 h, PLC/PRF/5 cells were incubated with medium containing virus along with 5 μg/mL polybrene. Two days post-infection, cells were selected in the medium containing 2 μg/mL puromycin and then single-cell colonies were selected after seeding in 96-well plates. For genotyping, clonal cell genomic DNA was extracted with a Genomic DNA Purification Kit (Genloci Biotechnologies Inc., Jiangsu, China) and then cloned into the pMD19-T TA cloning vector (Takara, Kyoto, Japan) for sequencing. PCK1-knockout efficiency was confirmed by western blotting. PCK1-knockout and control cells are hereafter referred to as PCK1-KO and parental cells, respectively. All primers are listed in Additional file 1: Table S1.

### Proliferation assay

Cells were seeded in 96-well plates at a density of 1 × 10^3^ cells/well and cultured for 5 days. The absorbance at 490 nm was measured in real time every 24 h after incubation with 20 μL of 3-(4,5-dimethylthiazol-2-yl)-5-(3-carboxymethoxyphenyl)-2-(4-sulfophenyl)-2H-tetrazolium (MTS) labeling reagent solution (Promega) for 2 h.

### Colony formation assay

Cells (1 × 10^3^ cells/well) were seeded in 6-well plates and cultured at 37 °C and 5% CO_2_ for 14 days. The medium was replaced every three days. Cell colonies were washed twice with PBS, fixed with 4% paraformaldehyde for 30 min, and stained with 0.1% crystal violet for 20 min. The experiment was repeated at least three times.

### Cell cycle analysis

Cells were harvested at a density of 1.0 × 10^6^ cells/mL, washed with PBS, and fixed with 70% ethanol at 4 °C overnight. Then, the cells were washed with PBS, stained with propidium iodide, and subjected to cell cycle analysis using a FACSCalibur instrument (BD Biosciences, Franklin Lakes, NJ, USA) and CellQuest software.

### Determination of intracellular ATP levels

Cellular ATP production was detected using an ATP Assay Kit (Beyotime) according to manufacturer’s instructions [18]. Briefly, cells were collected in a 1.5-mL tube and centrifuged. Then, 200 μL of lysis buffer from the ATP Assay Kit was added to each tube, after which the lysates were centrifuged at 12,000×*g* for 5 min. ATP production was measured by a luciferase assay of cell lysates and normalized to cellular protein concentrations (nM ATP/mg protein). Protein levels of the supernatant were measured at 562 nm with a BCA assay kit (Beyotime).

### Animal models

For the subcutaneous xenograft tumor model, 18 male BALB/c nude mice (5–6 weeks of age) were randomly divided into three groups. MHCC-97H cells were mock-infected or infected with AdGFP or AdPCK1 for 24 h, then collected for subcutaneous injection (1 × 10^5^ cells/injection) into the flanks of athymic BALB/c nude mice. Tumor volume was monitored by measuring the length (L) and width (W) at 3-day intervals for 5 weeks. Tumor volume [cm^3^] was calculated as L [cm] × (square of W [cm^2^])/2. After 5 weeks, the mice were sacrificed and tumor tissues were collected for histological analysis.

For the orthotopic implantation model, 15 BALB/c nude mice were randomly divided into parental, PCK1-KO, and metformin-treated PCK1-KO groups (five mice per group). The PLC/PRF/5 parental and PCK1-KO cells (1 × 10^5^ cells/injection) were collected and implanted into the left lobes of nude mice livers. On day 7 after implantation, the mice were treated with metformin (250 mg/kg per day, intraperitoneally) or PBS (equal volume, intraperitoneally) for 6 weeks. One mouse in a treatment group died due to postoperative infection during the experiment. Seven weeks after implantation, the mice were sacrificed and liver tissues were collected for histological examination.

All animal experiments were carried out according to the guidelines of the Institutional Animal Care and Use Committee at Chongqing Medical University (project license number: 2017012) and animal care and use protocols adhered to national regulations for the administration of laboratory animals.

### Statistical analysis

Data are expressed as the mean ± standard deviation (SD). Means were compared using Student’s *t*-test when comparing two groups or one-way analysis of variance (ANOVA) when comparing more than two groups. Correlations were assessed using the Spearman Rank Correlation test. Two-sided *P* values < 0.05 were considered statistically significant. Statistical analyses were conducted using GraphPad Prism 7.0 software (La Jolla, CA, USA).

### Nucleotide sequence accession numbers

The RNAseq data generated in this study have been submitted to the NCBI GEO database (http://www.ncbi.nlm.nih.gov/geo) under the identifier GSE117822.

## Results

### PCK1 suppresses hepatoma cell proliferation via G1/S phase cell cycle arrest

**Fig. 1:**
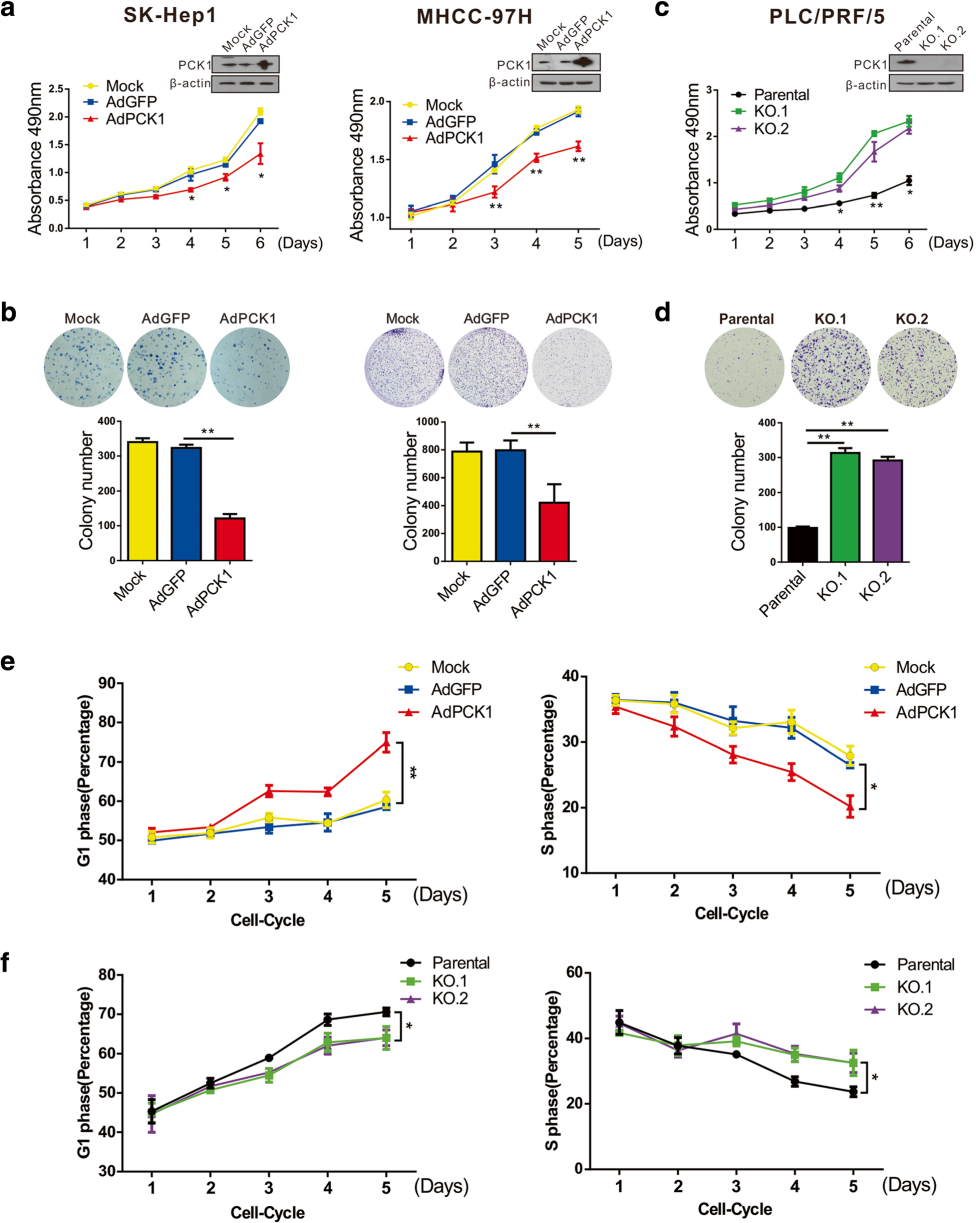
Suppression of PCK1 expression promotes cell proliferation by inducing G1/S phase transition in hepatoma cells. **a**-**d** Cell proliferation curves and colony formation assay. **a**, **b** SK-Hep1 and MHCC-97H cells were infected with adenoviruses expressing PCK1 or GFP control. PCK1 overexpression (PCK1-OE) was confirmed by western blotting. **c**, **d** Endogenous PCK1 was knocked out in PRF/PLC/5 cells by CRISPR-Cas9 and confirmed by western blotting. Cells were seeded into 96-well plates at 1 × 10^3^ cells/mL and counted every 24 h in triplicate for cell proliferation analysis. Cells were seeded in 6-well plates at 1 × 10^3^ cells/well and cultured for 2 weeks for colony formation assays. **e, f** Flow cytometric analysis. **e** PCK1-OE SK-Hep1 cells and **f** PCK1-KO cells were collected at different time points and subjected to flow cytometry. Populations of cells at G1 and S phases are indicated as percentages of the whole cell population. Data represent the mean ± SD of three independent experiments; (means ± SD; *P < 0.05, **P < 0.01)

Previous studies have indicated that PCK1 expression is significantly lower in tumor tissues than in normal liver tissues and that downregulated PCK1 expression correlates with poor prognosis [18, 19]. To clarify the function of PCK1 during carcinogenesis and progression of HCC, we first examined endogenous PCK1 levels in several hepatoma cell lines and MiHA cells. We found strong endogenous PCK1 expression in PLC/PRF/5, HepG2, and MiHA, modest expression in Huh7, and low expression levels in SK-Hep1 and MHCC-97H cells (Additional file 2: Figure S1a). According to the endogenous PCK1 expression pattern in hepatoma cells, we evaluated the effect of PCK1-overexpression (OE) in SK-Hep1, Huh7, and MHCC-97H cells as well as the effect of PCK1-knockout (KO) in PLC/PRF/5 cells. Overexpression of PCK1 significantly suppressed proliferation of SK-Hep1, MHCC-97H, and Huh7 cells compared with that of GFP-expressing control cells (Fig. 1a, b, and Additional file 2: Figure S1b, S1c). On the other hand, PCK1 knockout promoted cell proliferation in PLC/PRF/5 cells (Fig. 1c and d).

To determine whether the impact of PCK1 on cell proliferation is associated with cell cycle control, we performed flow cytometric analyses of PCK1-overexpression (OE) or PCK1-knockout (KO) hepatoma cells. Cells were cultured in normal DMEM and collected continuously during days 1–5 post-synchronization. Time-course analysis indicated that PCK1 overexpression significantly inhibited G1/S transition in SK-Hep1 cells after prolonged incubation, especially 3–5 days after synchronization treatment (Fig. 1e and Additional file 3: Figure S2a), whereas PCK1 deficiency promoted G1/S transition in PLC/PRF/5 cells 3–5 days post-synchronization (Fig. 1f and Additional file 3: Figure S2b). Taken together, these results indicate that PCK1 inhibits hepatoma cell proliferation by delaying G1/S transition.

### PCK1 inhibits the CDK/Rb/E2F signaling pathway

**Fig. 2:**
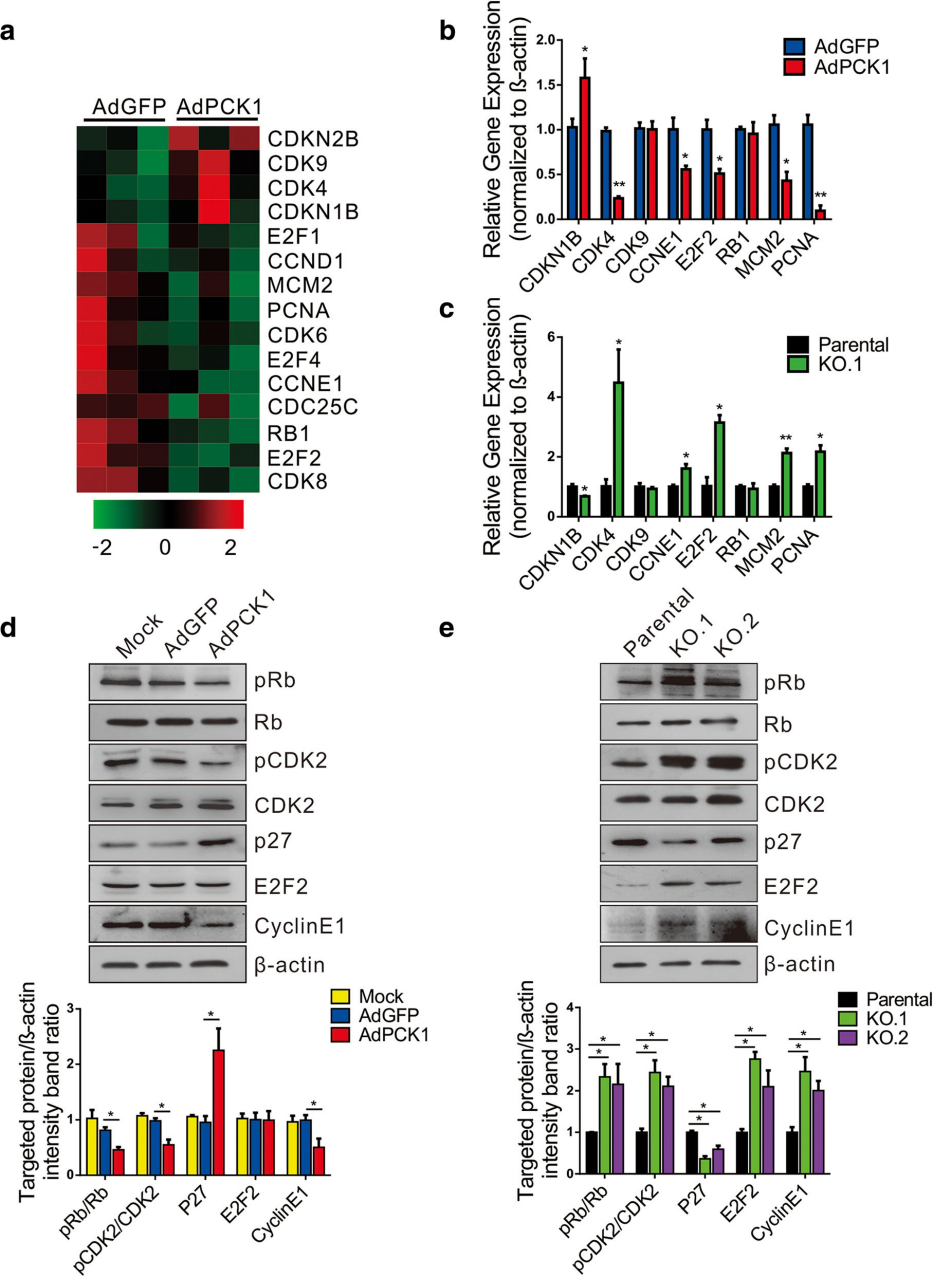
PCK1 controls the expression of cell cycle-related genes. **a** Heat map representing the differential expression of E2F family members, transcription factors, and E2F target genes as determined by RNA-Seq in PCK1-OE and GFP control cells. Hepatoma cells infected with adenoviruses expressing PCK1 (AdPCK1) or vector control (AdGFP) were subjected to RNA-Seq analysis. **b**–**e** Differentially expressed genes (DEGs) were validated in PCK1-OE and PCK1-KO hepatoma cells by RT-qPCR and western blot analysis. PCK1-OE SK-Hep1 cells and PCK1-KO cells were treated as described in Fig. 1. **b**, **c** mRNA expression levels of the indicated genes were measured by qRT-PCR. **d**, **e** Western blot analysis of the indicated genes in PCK1-OE and PCK1-KO cells. β-actin was used as a loading control. Relative levels of target proteins were measured by densitometry. Data are presented as the mean ± SD of three independent experiments; *P < 0.05, **P < 0.01

To explore the mechanism underlying PCK1-induced G1 cell cycle arrest, we performed genome-wide RNA-Seq of Huh7 cells infected with AdPCK1 or AdGFP control (Fig. 2). Analysis of the differentially expressed genes (DEGs) indicated that multiple inhibitors of cell cycle progression, especially G1-S phase inhibitors including CDKN2B (p15), CDKN1B (p27), CDK4, and CDK9, were strongly upregulated by PCK1. Meanwhile, PCK1 dramatically downregulated the cell cycle accelerators E2F2, CCNE1, and CCND1 (Fig. 2a). These RNA-seq results were validated by qRT-PCR. mRNA levels of CDK4, MCM2, PCNA, CCNE1, and E2F2 were found downregulated, whereas those of CDKN1B (p27^Kip1^) were enhanced by PCK1. In contrast, PCK1 knockout resulted in reversed regulatory effects on these molecules (Fig. 2b and c).

Next, we conducted immunoblot analysis to determine the impact of PCK1 on the protein levels of cell cycle-related proteins, especially G1/S cell cycle checkpoint-related proteins. We found that the p27^Kip1^ protein was dramatically upregulated in AdPCK1-infected SK-Hep1 cells, whereas pRB, pCDK2, and cyclin E1 were markedly downregulated in PCK1-OE cells (Fig. 2d). In contrast, PCK1 knockout increased the protein levels of pRB, pCDK2, E2F2, and cyclin E1 in PLC/PRF/5 cells, whereas p27^Kip1^ expression was significantly decreased (Fig. 2e). Together, these findings suggest that PCK1 may dampen CDK/Rb/E2F signaling to inhibit cell cycle progression, resulting in the suppression of cell proliferation.

### PCK1 arrests the cell cycle through AMPK activation

**Fig. 3:**
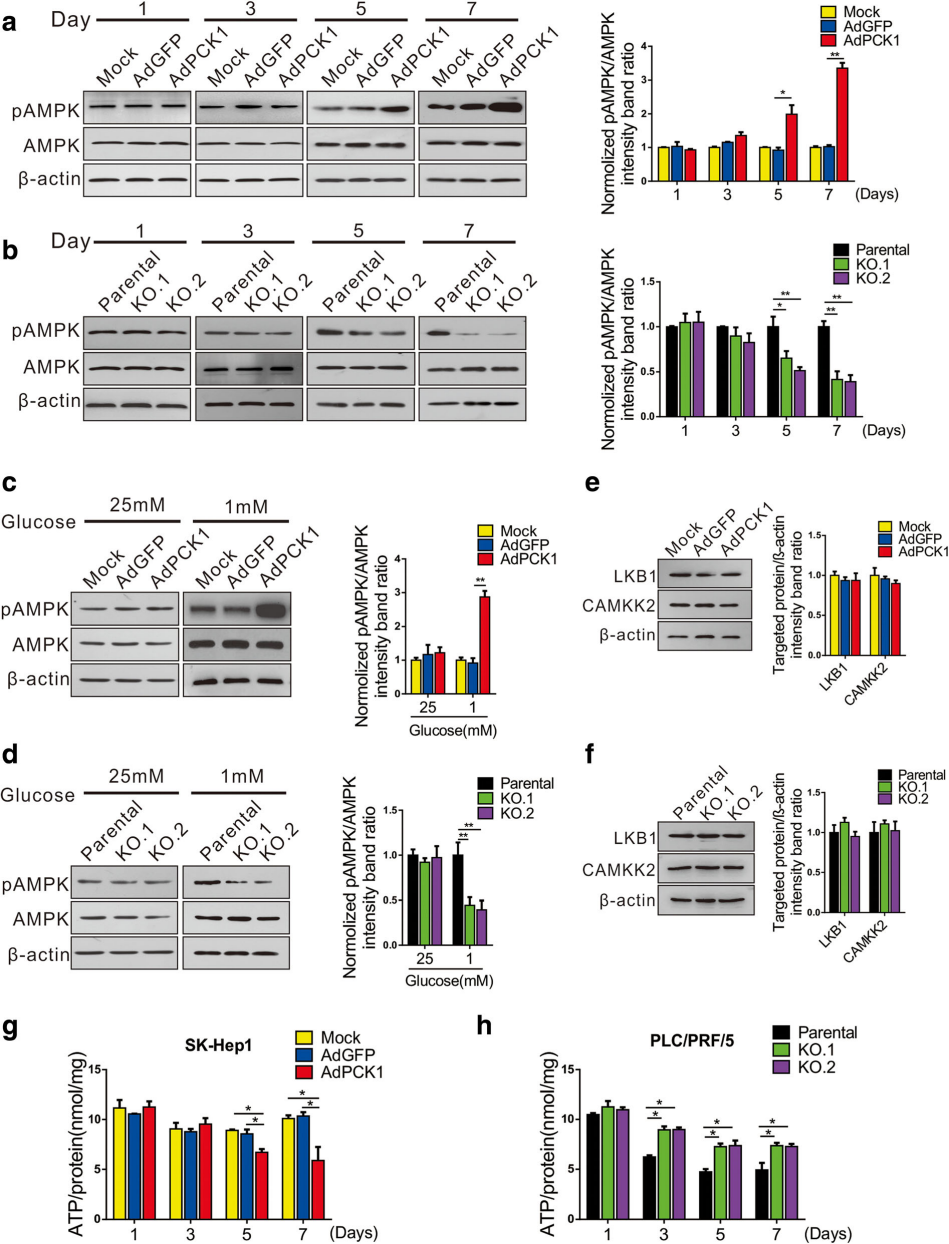
PCK1 activates AMPK by decreasing ATP levels in glucose-deprived hepatoma cells. **a**–**d** Immunoblot analysis of AMPK and phosphorylated AMPK. (**a**) PCK1-OE or (**b**) PCK1-KO hepatoma cells were cultured in normal DMEM for 1, 3, 5, or 7 days, while (**c**) PCK1-OE or (**d**) PCK1-KO hepatoma cells were cultured in DMEM containing different concentrations of glucose (25 or 1 mM) for 12 h. AMPK phosphorylation was measured by western blot analysis. Results are representative of three independent experiments. **e**, **f** Protein expression of LKB1 and CaMKK2 in (**e**) PCK1-OE and (**f**) PCK1-KO hepatoma cells. β-actin was used as a loading control. Integrated density of pAMPK/AMPK, LKB1, and CaMKK2 was quantitatively analyzed using ImageJ software and the results were normalized to mock or parental group. *P < 0.05, **P < 0.01. (**g**, **h**) Quantification of ATP concentrations in (**g**) PCK1-OE and (**h**) PCK1-KO HCC cells cultured in normal DMEM medium for 1, 3, 5, or 7 days. Cellular ATP levels were determined as described in the Methods section. Values represent mean ATP/protein levels ± SD of triplicate samples. *P < 0.05, **P < 0.01

PCK1 catalyzes the formation of PEP from OAA, this reaction—along with GTP consumption—may result in cellular energy reduction [20]. Therefore, we evaluated whether AMPK, an important cellular energy sensor, is involved in cell cycle regulation by PCK1. We first monitored the protein levels of AMPK and phospho-AMPK (Thr 172; pAMPK) in PCK1-OE and PCK1-KO hepatoma cells under normal culture conditions at different time points. pAMPK levels were significantly upregulated in PCK1-OE cells after 5-day culture, with higher levels of pAMPK detected after 7-day culture. (Fig. 3a). Conversely, knockout of PCK1 in PRF/PLC/5 cells inhibited pAMPK expression after 5 or 7-day cultures compared with that of parental cells (Fig. 3b). Considering that nutrients such as glucose in the culture medium are gradually consumed during long-term culture, we compared AMPK and pAMPK expression levels in PCK1-OE and PCK1-KO hepatoma cells cultured under low-glucose (1 mM) or normal-glucose (25 mM) conditions for 12 h. Consistent with the AMPK expression profile in long-term cell culture, PCK1 overexpression markedly upregulated pAMPK expression (Fig. 3c), whereas knockout of PCK1 downregulated pAMPK expression (Fig. 3d) in cells under low-glucose conditions.

AMPK is mainly activated via phosphorylation through the increased expression of liver kinase B1 (LKB1), the Ca^2+^/calmodulin-dependent protein kinase kinase2 (CaMKK2), and an elevation in cellular AMP/ATP ratios [21]. Therefore, we further explored the mechanism responsible for AMPK activation by PCK1. Immunoblot assays showed that protein levels of LKB1 and CaMKK2 were not affected upon modulation of PCK1 expression (Fig. 3e and f), indicating that LKB1 and CaMKK2 are not largely responsible for PCK1-mediated AMPK activation. However, ATP levels in PCK1-OE SK-Hep1 cells were significantly reduced after 5 and 7-day culture (Fig. 3g) and markedly increased in PCK1-KO PLC/PRF/5 cells after 3, 5, and 7-day culture (Fig. 3h), which is highly consistent with the time-course analysis of AMPK activation. These results indicate that a decrease in ATP generation plays an essential role in PCK1-induced AMPK activation.

### PCK1 delays G1/S phase transition by upregulating p27^Kip1^ expression via AMPK activation

**Fig. 4:**
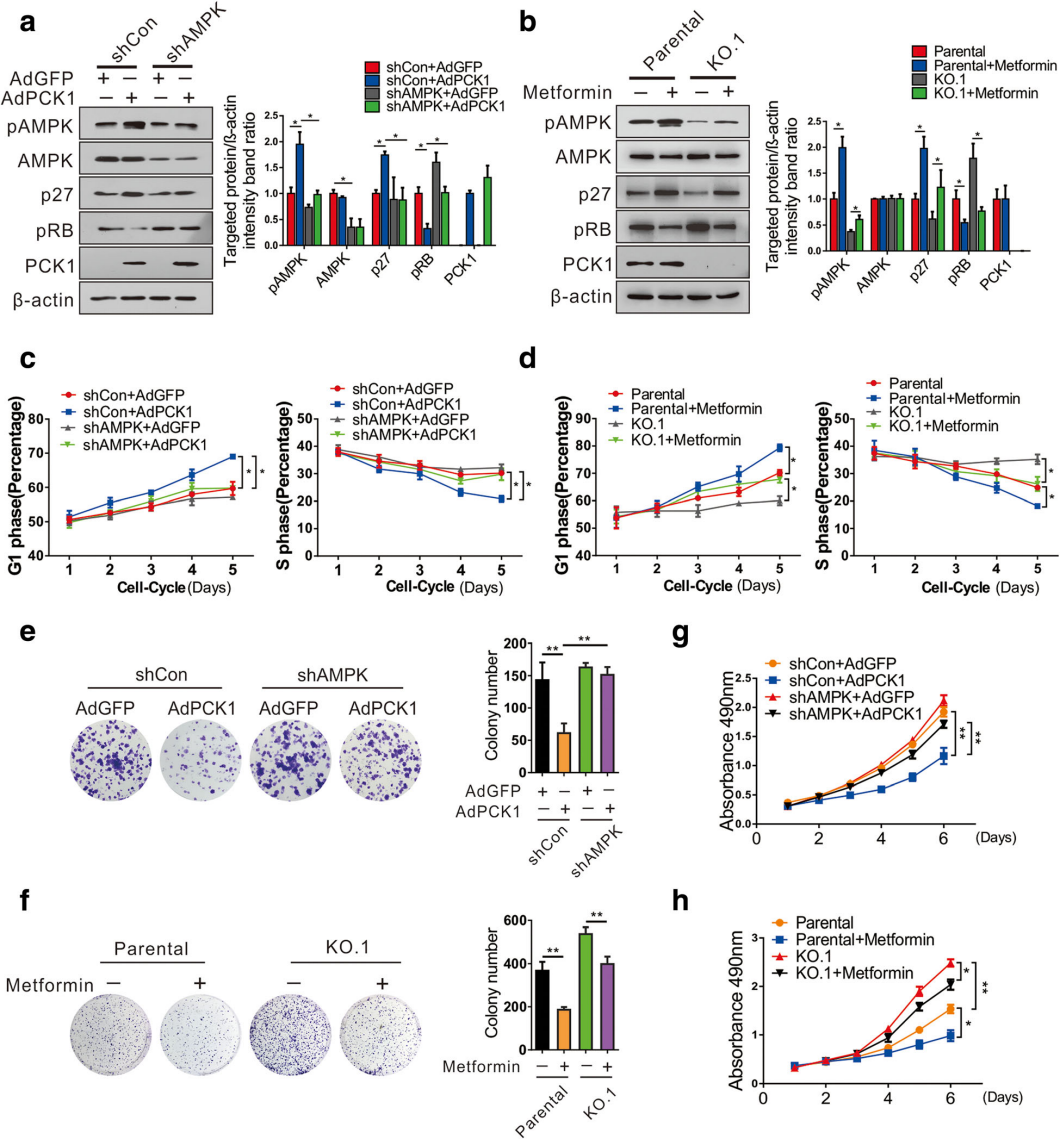
PCK1-mediated AMPK activation and delayed cell proliferation depend on p27^Kip1^ expression and phosphorylation of Rb protein. **a**, **b** Protein levels of the indicated proteins in PCK1-OE and KO hepatoma cells. β-actin was used as a loading control. **a** AMPK-stable knockdown SK-Hep1 cells (SK-Hep1/shAMPK) or control cells (SK-Hep1/shControl) were infected with AdPCK1 or AdGFP for 48 h, respectively. **b** PCK1-KO or parental PLC/PRF/5 cells were treated with 5 mM metformin for 12 h. Cell lysates were then subjected to immunoblot assays with the indicated antibodies for detecting the protein expression levels of p27^Kip1^, pRB, AMPK, and pAMPK. Results shown are representative samples from at least three independent experiments. Integrated density of these proteins was quantitatively analyzed and the results normalized to the shCon+AdGFP or parental group. **c**, **d** Flow cytometric analysis. Cells were treated as described above, collected at different timepoints (1, 2, 3, 4, or 5 days), and subjected to flow cytometry. Percentages of cells in the G1 and S phases are shown within each graph. Data represent the mean ± SD of three independent experiments. *P < 0.05. **e**–**h** Cell proliferation curves and colony formation assay. SK-Hep1 cells infected with shAMPK or shControl lentivirus were treated as described in **a** while PCK1-KO and parental PLC/PRF/5 cells were treated with 0.5 mM metformin. **e**, **f** Representative images and quantification of colony formation. Cell colonies were counted after 2 weeks of incubation. **g**, **h** Cell proliferation curves. Cells were counted every 24 h in triplicate. Values represent the mean ± SD of three independent experiments. *P < 0.05, **P < 0.01

Considering that AMPK activation is involved in PCK1-induced growth inhibition, we explored whether knockdown of AMPK can partially reverse the cell cycle arrest and growth-retarding effects of PCK1. We suppressed AMPK expression by infecting SK-Hep1 cells with lentiviruses expressing shRNAs targeting AMPK (shAMPK1# and shAMPK2#; Additional file 4: Figure S3a). As expected, AMPK silencing strongly decreased p27^Kip1^ expression and increased pRb expression under low-glucose conditions (1 mM glucose; Additional file 4: Figure S3b). In addition, knockdown of AMPK prevented PCK1-induced upregulation of p27^Kip1^, downregulation of pRb, delay of G1/S transition, and inhibition of cell proliferation (Fig. 4, and Additional file 5: Figure S4a). The AMPK activator metformin partially offset the PCK1 deficiency-mediated downregulation of p27^Kip1^, upregulation of pRb, promotion of G1/S transition, and acceleration of hepatoma cell proliferation (Fig. 4, and Additional file 5: Figure S4b). These results indicate that PCK1 induces p27^Kip1^ expression through activation of the AMPK pathway, resulting in suppression of the G1/S transition and eventual inhibition of hepatoma cell proliferation.

### PCK1 acts as a tumor suppressor of HCC in vivo

**Fig. 5:**
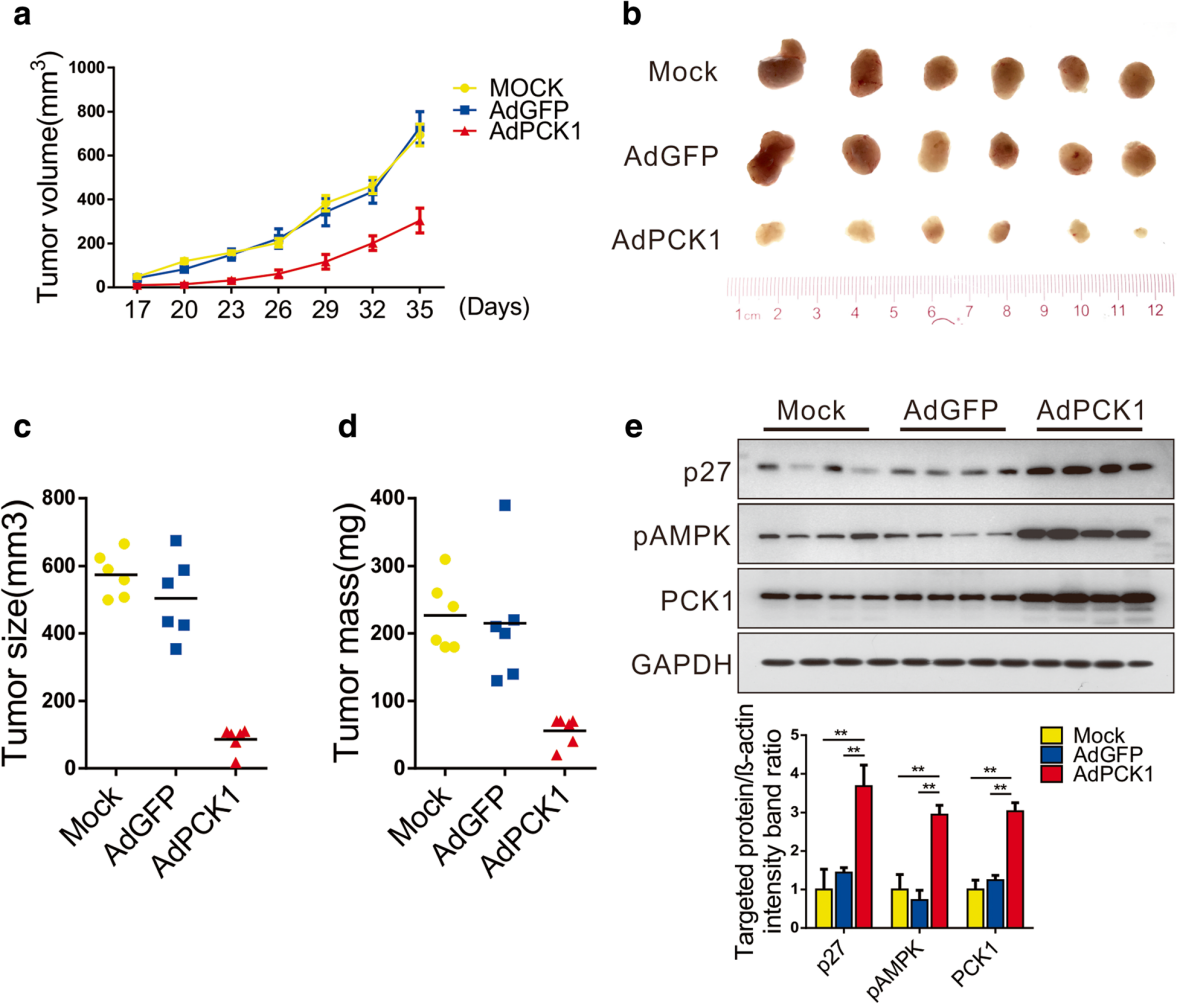
PCK1 inhibits hepatoma growth in a subcutaneous xenograft mouse model. MHCC-97H cells were infected with AdGFP or AdPCK1 for 24 h. Control MHCC-97H cells and infected cells (1 × 10^5^ cells/injection) were injected into the flank of nude mice (*n* = 6 per group). **a** Tumor growth curves of subcutaneous xenograft tumor model mice. Tumor volumes were calculated as M × (square of W)/2. *P < 0.05 by two-tailed unpaired Student’s *t*-test. Mice were sacrificed 38 days after injection, followed by xenograft tumor dissection. **b** Representative images of xenografts tumors. **c** Tumor size and **d** tumor weight of subcutaneous xenografts. **e** PCK1, pAMPK, and p27^Kip1^ protein expression in tumor tissue samples as detected by western blotting. Relative levels of the indicated proteins were quantified using ImageJ software. **P < 0.01

Next, we evaluated the effect of PCK1 gain-of-function on hepatoma cell growth in a murine subcutaneous xenograft model. As shown in Fig. 5a, b, and Additional file 6: Figure S5a, tumor growth was significantly suppressed in nude mice that had received PCK1-OE cells. Furthermore, tumor volume and weight were notably reduced in the PCK1-OE group compared with the GFP control group (Fig. 5c and d). Moreover, pAMPK and p27^Kip1^ expression was markedly increased in tumor tissues exogenously expressing PCK1, as evidenced by western blotting and IHC (Fig. 5e and Additional file 6: Figure S5b). Collectively, these findings indicate that PCK1 inhibits tumor growth both in vitro and in vivo.

### Metformin-mediated AMPK activation suppresses liver tumor growth in nude mice

**Fig. 6:**
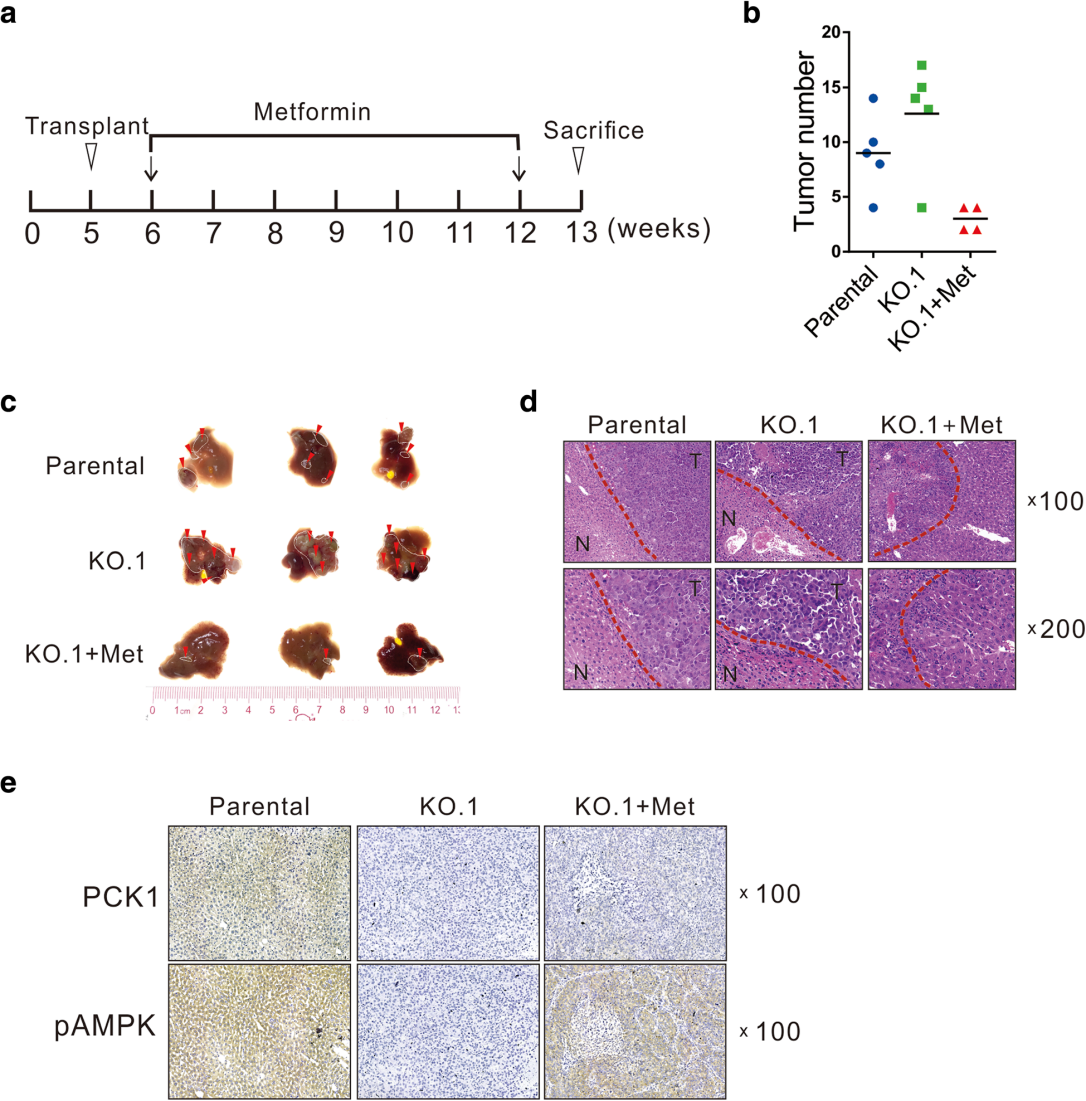
Metformin suppresses tumor growth in an orthotopic implantation tumor model established with PCK1-KO hepatoma cells. PCK1-KO and parental cells (1 × 10^5^ cells/injection) were implanted into the left lobes of nude mice livers (*n* = 5 per group). One week after implantation, mice were treated with metformin (250 mg/kg per day, intraperitoneally) or PBS (equal volume, intraperitoneally) for 6 weeks. The mice were sacrificed 7 weeks after implantation. **a** Schematic overview of the experiment design. **b** Tumor foci number in the liver of each mouse. **c** Representative gross appearance of murine livers. Red arrows: metastatic foci. **d**, **e** Representative **d** HE staining and **e** IHC detection of PCK1 and pAMPK in liver tumor tissues. Magnification: 100×, 200×

Considering that the murine subcutaneous xenograft model using PLC/PRF/5 cell is difficult to achieve, we examined the effects of PCK1 deficiency on tumor growth in an orthotopic transplantation HCC mouse model. PCK1-KO and parental PLC/PRF/5 cells were transplanted into the livers of nude mice. Compared with parental PLC/PRF/5 cells, PCK1-KO significantly promoted orthotopic tumor formation in mice (Fig. 6a–c).

To investigate whether metformin reverses the PCK1 deficiency-induced tumor-promoting effects, mice orthotopically implanted with PCK1-KO hepatoma cells were divided into two groups, one group was treated with metformin (250 mg/kg/day) and the other received PBS as control (Fig. 6a). We found that metformin treatment dramatically repressed tumor growth in mice (Fig. 6b and c). HE staining revealed that livers of the PCK1-KO group displayed many pleomorphic and atypical hepatocytes as well as remarkably altered nodular liver structure, which were attenuated by metformin treatment (Fig. 6d). Furthermore, pAMPK expression was significantly higher in the metformin-treated group than in the control group (Fig. 6e), and this result was consistent with our in vitro findings. Taken together, these results demonstrate that metformin treatment efficiently inhibits the tumorigenesis of PCK1-KO hepatoma cells in nude mice through the AMPK/p27^Kip1^ axis.

### Downregulation of PCK1 is positively correlated with pAMPK and p27^Kip1^ expression in HCC patients

**Fig. 7:**
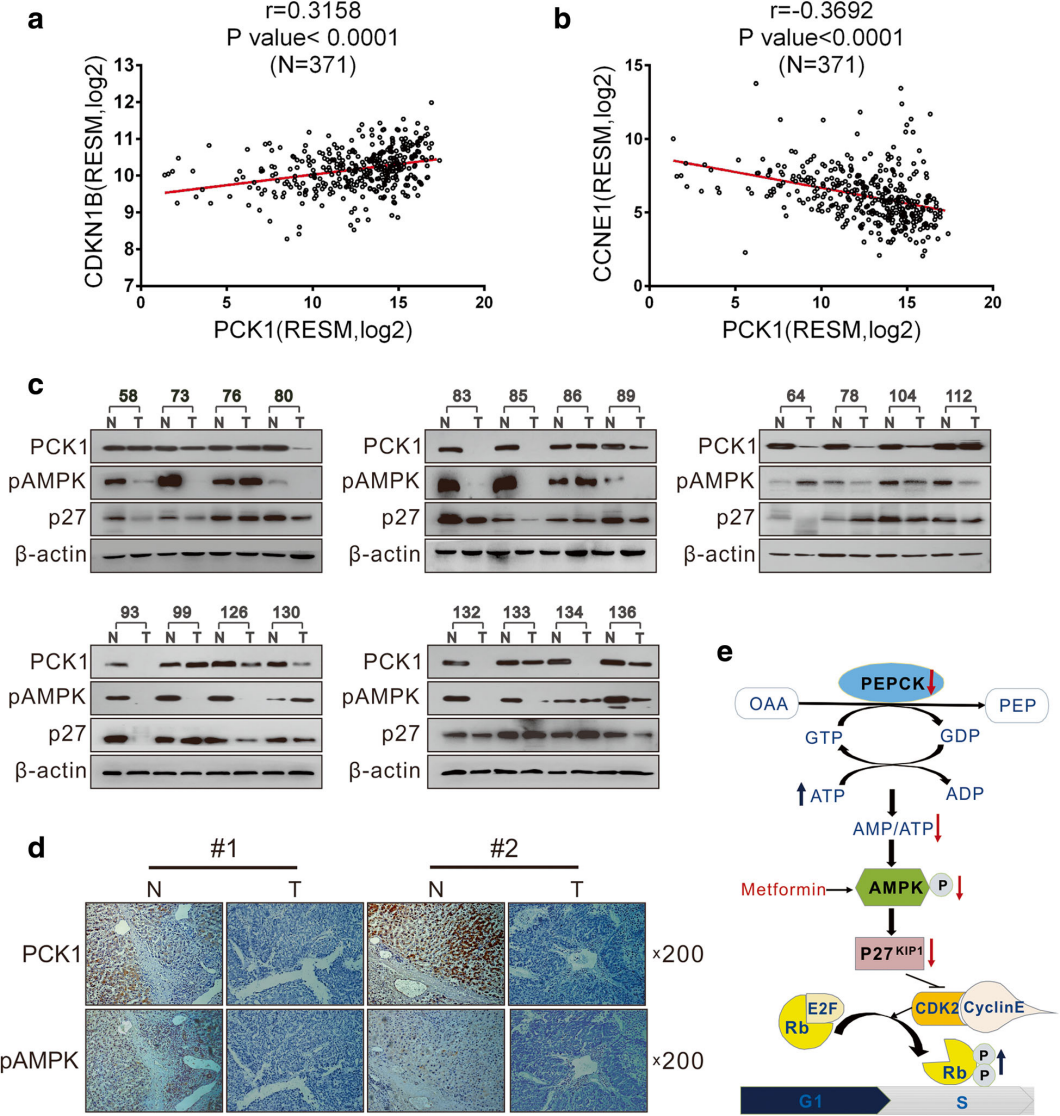
PCK1 expression is reduced in HCC tissues and is positively correlated with pAMPK and p27Kip1 expression. **a**, **b** Correlation between CDKN1B, CCNE1, and PCK1. Correlation between mRNA expression levels of **a** CDKN1B or **b** CCNE1 and PCK1 based on data from 371 HCC patients from the TCGA database mRNA dataset. Statistical analysis was performed using Pearson’s correlation coefficient (*P* < 0.0001). **c** PCK1, pAMPK, and p27^Kip1^ protein levels in 20 paired primary HCC tissues and adjacent non-tumor tissues. β-actin was used as a loading control. **d** Representative IHC images of PCK1 and pAMPK in HCC and adjacent non-tumor tissues. Magnification, 200×. **e** Proposed model for activation of AMPK and cell cycle arrest in PCK1-induced inhibition of hepatoma cell growth. The gluconeogenic enzyme PCK1 catalyzes an energy-consuming reaction, which consumes GTP to generate GDP. PCK1 induces AMPK activation via ATP-dependent mechanisms both in hepatoma cells and in vivo. AMPK activation further promotes G1/S transition by regulating p27^Kip1^ expression and negatively modulating pCDK2, cyclin E1, and pRB expression. Eventually, PCK1 inhibits the growth of hepatoma cells and the development of HCC through AMPK activation and cell cycle arrest

Finally, we analyzed PCK1 expression in clinical HCC samples and explored the potential association between PCK1 and p27^Kip1^ expression in HCC tissues. In an independent cohort of 371 HCC samples from the Cancer Genome Atlas (TCGA) database, PCK1 expression was positively correlated with p27^Kip1^ expression (*r* = 0.3158, *P* < 0.0001; Fig. 7a) and negatively correlated with CCNE1 expression (*r* = 0.3692, *P* < 0.0001; Fig. 7b). In addition, we examined the protein levels of PCK1, pAMPK, and p27^Kip1^ in paired HCC and adjacent non-cancerous tissues from 20 patients. PCK1 protein levels were markedly lower in 16 of the 20 HCC tissues (85%; Fig. 7c) compared with non-cancerous tissues. Furthermore, pAMPK and p27^Kip1^ levels were also reduced in 14 and 13 of the 16 PCK1-downregulated HCC tissues, respectively. (Fig. 7c). Downregulation of PCK1 and pAMPK expression in HCC tissues was confirmed by IHC staining (Fig. 7d). These results reveal that downregulation of PCK1 is linked to decreased levels of pAMPK and p27^Kip1^ in HCC tissues, suggesting that PCK1 deficiency may be involved in HCC progression through inhibition of the AMPK pathway and repression of p27^Kip1^ expression.

## Discussion

Hepatic gluconeogenesis is an enzymatic process that ensures glucose production rates exactly match whole-body glucose requirements; PCK1 is regarded as one of the key enzymes involved in this process [22]. Recent studies have shown that PCK1 functions as a regulator of hepatic energy metabolism and gluconeogenesis, with its dysregulation linked to metabolic diseases, such as diabetes, obesity, insulin resistance, and tumor development [23–25]. However, PCK1 has contrasting effects on tumorigenesis in different tumor types. In colorectal cancer and melanoma, increased PCK1 expression is associated with upregulated anabolic metabolism and cell proliferation, indicating that PCK1 functions as an oncogene in these cancers [20, 25, 26]. In contrast, Zeribe et al. [27] observed that PCK1 ranked in the top 25 downregulated genes in at least six different HCC datasets. Similarly, PCK1 downregulation has been observed in other cohorts of HCC, where restoration of its expression leads to tumor suppression [18, 19, 28], indicating that PCK1 acts as a tumor suppressor in HCC. However, the mechanisms underlying the crosstalk of PCK1 with oncogenic pathways in the modulation of cellular behavior, such as cell apoptosis and proliferation, remain poorly understood.

The present study demonstrated that PCK1 deficiency promotes HCC growth both in vitro and in vivo*,* whereas overexpression of PCK1 represses HCC growth, which is consistent with previous findings [18, 29]. Furthermore, transcriptome and cell cycle analysis revealed that PCK1 may suppress G1/S transition and cell proliferation in hepatoma cells via inhibiting the CDK/Rb/E2F signaling pathway. Mechanistically, PCK1 decreases cellular ATP levels and activates the AMPK/p27^Kip1^ axis in glucose-deprived hepatoma cells. Interestingly, metformin, an AMPK activator, restrains the tumorigenesis of PCK1-deficient HCC. Based on our findings, we suggest a potential model for AMPK activation and cell cycle arrest in the PCK1-induced inhibition of hepatoma cell proliferation (Fig. 7e).

AMPK is recognized as a cellular energy sensor that is activated by phosphorylation at Thr172 [30]. Once activated, AMPK triggers catabolic pathways to generate ATP, while inhibiting anabolic ATP-consuming pathways in an attempt to restore cellular energy homeostasis [13, 31, 32]. AMPK is known to inhibit gluconeogenesis by phosphorylating CREB-regulated transcription coactivator 2 [33] and class IIA histone deacetylases [34]—co-activators of the CREB and FOXO pathways, respectively [35]. Interestingly, a recent study reported that the glycolytic enzymes aldolase and fructose-1,6-bisphosphate can suppress AMPK activation [36]. The present study demonstrated that the gluconeogenic enzyme PCK1 also plays an important role in AMPK activation. As previously reported, AMP binding to AMPK γ-subunits causes AMPK allosteric activation [37, 38], while upstream kinases such as LKB1 [39] and CaMKK2 [40] activate AMPK through modulating the phosphorylation state of AMPK α-subunit at Thr172 [21]. Our study showed that upon modulation of PCK1 expression, protein levels of LKB1 and CaMKK2 were not affected while ATP levels were significantly altered. On the other hand, PCK1 was reported to consume GTP when catalyzing the formation of PEP from OAA [41]. Taking into consideration the conversion of GTP to ATP via the nucleoside diphosphate kinase reaction [42, 43], PCK1 may be involved in the regulation of intracellular ATP levels. In support of our findings, Liu et al. [18] recently reported that PCK1 activated AMPK upon glucose deprivation in HCC. Their study revealed that forced PCK1 expression induced energy crisis and oxidative stress, leading to cell apoptosis under low-glucose conditions; however, the direct consequences of AMPK activation were not illuminated in depth. Here, we demonstrated that PCK1-induced AMPK activation increases p27^Kip1^ expression and suppresses hepatoma cell proliferation. Several studies have suggested that AMPK arrests cell cycle progression through the upregulation of G1/S phase transition inhibitors such as p27^Kip1^ [16, 44, 45]. Activation of AMPK may upregulate p27^Kip1^ expression directly or through the AMPK-p53-p27 axis. [15, 16] Moreover, p27 ^Kip1^ stability via phosphorylation is also increased by activation of AMPK signaling [44].

Metformin, a lipophilic biguanide, is used in first-line pharmacotherapy for type 2 diabetes. Increasing evidence suggests that it may also function as an anti-tumor drug. Metformin reportedly inhibits tumor development, progression, and metastasis by reducing cell proliferation [46], reversing epithelial-mesenchymal transition [47], and enhancing cell sensitivity to chemotherapeutic drugs [48]. The anti-tumor effect of metformin has been verified in breast, lung, and gastric cancers [46, 49, 50]. A recent study indicated that type 2 diabetes is correlated with an increased risk of HCC and that metformin decreases HCC risk in diabetic patients in a dose-dependent manner [51]. Our study corroborated the anti-tumor effect of metformin on HCC from the metabolic perspective.

## Conclusions

The present study revealed that the gluconeogenic enzyme PCK1 negatively regulates cell cycle progression and cellular proliferation via the AMPK/p27^Kip1^ axis, indicating a tumor suppressor role for PCK1 in HCC. Notably, the AMPK activator metformin restrained the tumorigenesis of PCK1-deficient HCC in vitro and in vivo, highlighting its potential therapeutic and protective effects on HCC. Our findings provide new insights into the mechanism of uncontrolled cell proliferation mediated by altered metabolism in liver cancer. Future studies on PCK1 dysregulation and hepatocarcinogenesis will provide novel diagnostic and therapeutic strategies against HCC.

## Additional files

Additional file 1: Primer sequences of target genes. (XLSX 13 kb)

Additional file 2: PCK1 supresses Huh7 cells proliferation. (JPG 2932 kb)

Additional file 3: Knockout of PCK1 promotes G1/S phase transition in hepatoma cells. (JPG 4192 kb)

Additional file 4: AMPK regulates the expression of p27 and pRB in glucose-deprived hepatoma cells. (JPG 2140 kb)

Additional file 5: PCK1 retards G1/S phase transition via AMPK phosphorylation. (JPG 4500 kb)

Additional file 6: PCK1 inhibits hepatoma growth in a subcutaneous xenograft mouse model. (JPG 3699 kb)

## Abbreviations

AMPK: 5’-AMP-activated protein kinase
ATP: Adenosine triphosphate
CDKs: Cyclin-dependent kinases
HCC: Hepatocellular carcinoma
PCK1: Phosphoenolpyruvate carboxykinase 1
Rb: Retinoblastoma protein
