# Supplementary figures and images for: PCK1 negatively regulates cell cycle progression and hepatoma cell proliferation via the AMPK/p27Kip1 axis
Source: J Exp Clin Cancer Res. 2019 Feb 4;38:50. doi: 10.1186/s13046-019-1029-y (PMC6360696; doi:10.1186/s13046-019-1029-y)

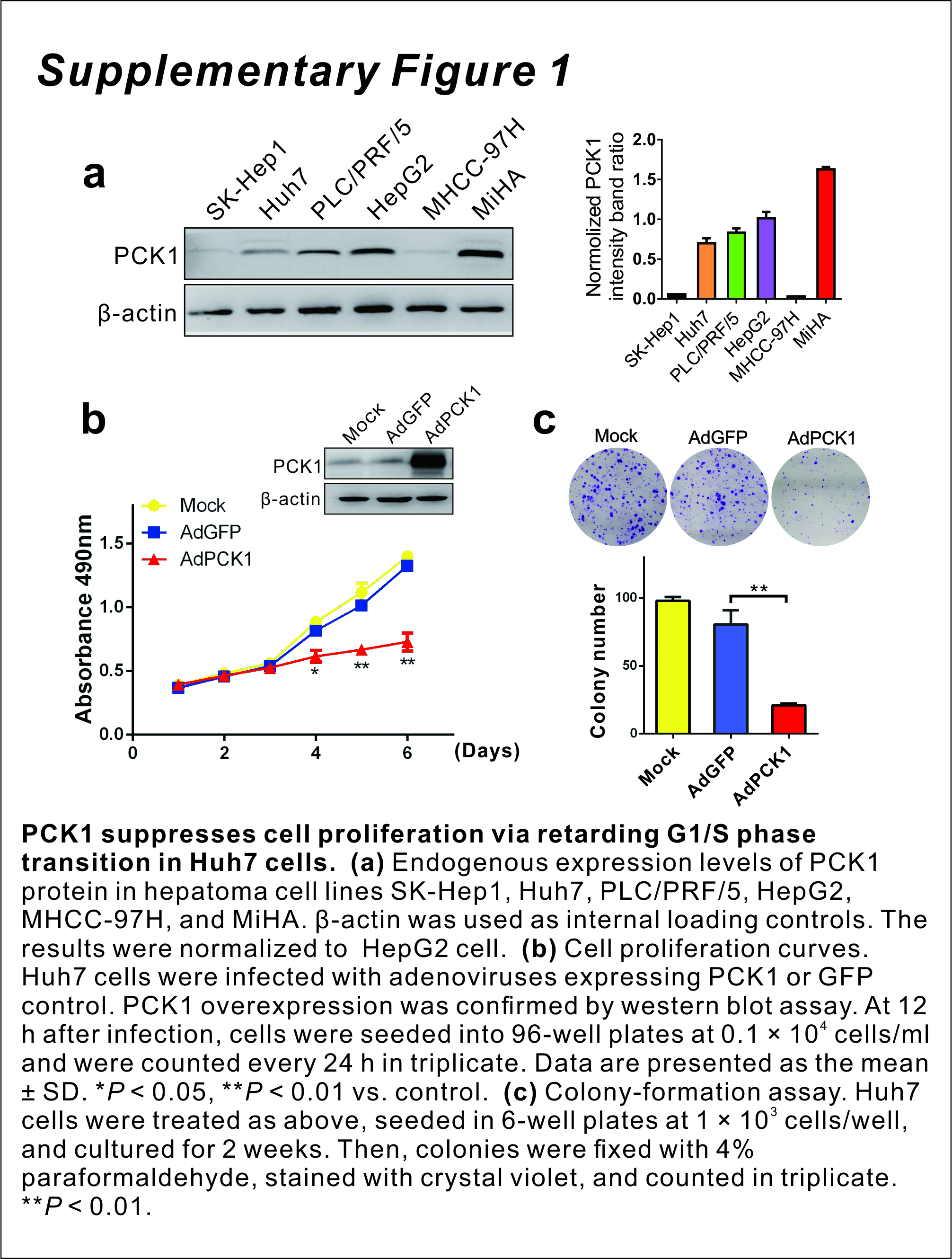

Supplement: Supplementary file 2 — PCK1 supresses Huh7 cells proliferation. (JPG 2932 kb) [file 13046_2019_1029_MOESM2_ESM.jpg]

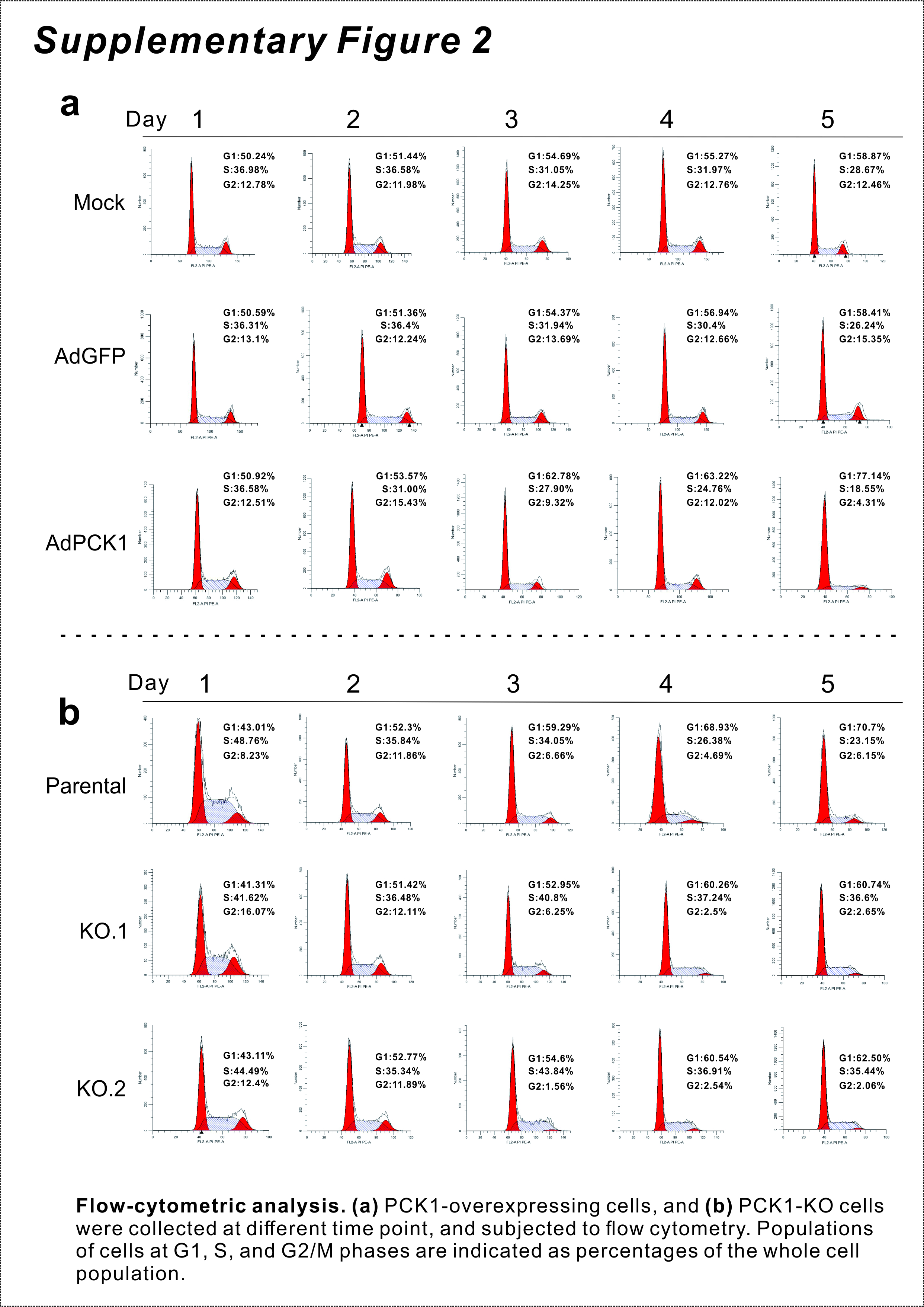

Supplement: Supplementary file 3 — Knockout of PCK1 promotes G1/S phase transition in hepatoma cells. (JPG 4192 kb) [file 13046_2019_1029_MOESM3_ESM.jpg]

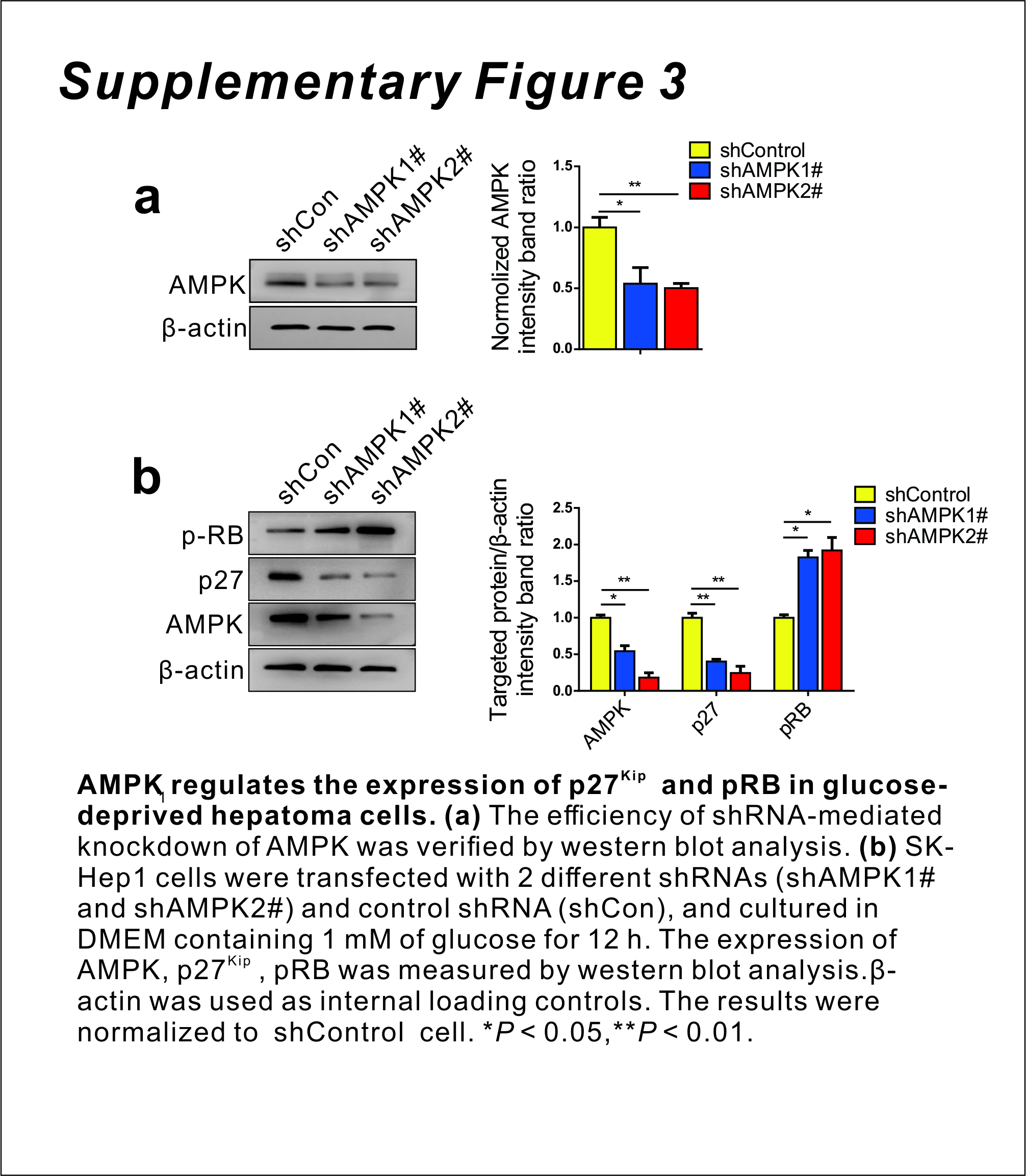

Supplement: Supplementary file 4 — AMPK regulates the expression of p27 and pRB in glucose-deprived hepatoma cells. (JPG 2140 kb) [file 13046_2019_1029_MOESM4_ESM.jpg]

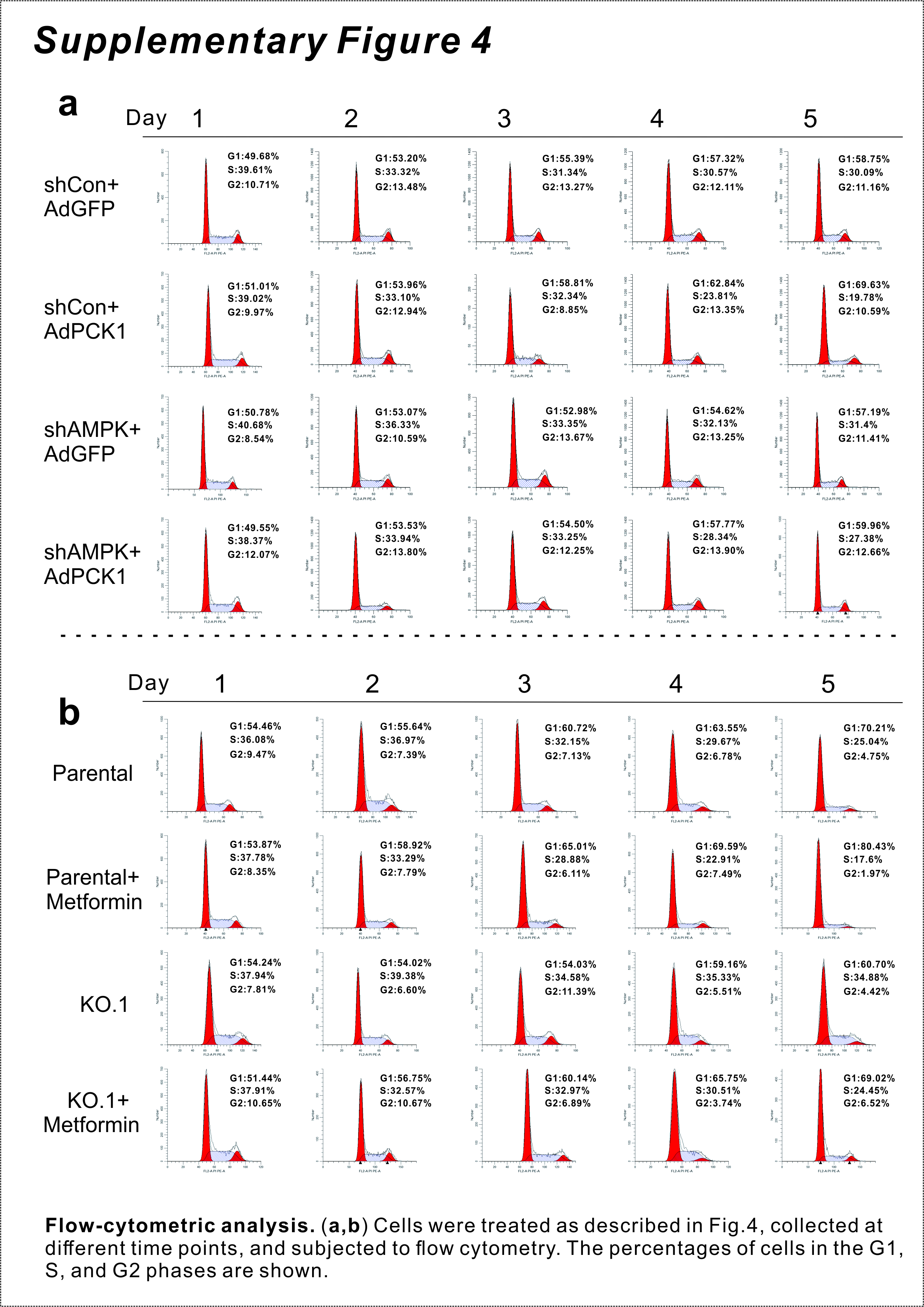

Supplement: Supplementary file 5 — PCK1 retards G1/S phase transition via AMPK phosphorylation. (JPG 4500 kb) [file 13046_2019_1029_MOESM5_ESM.jpg]

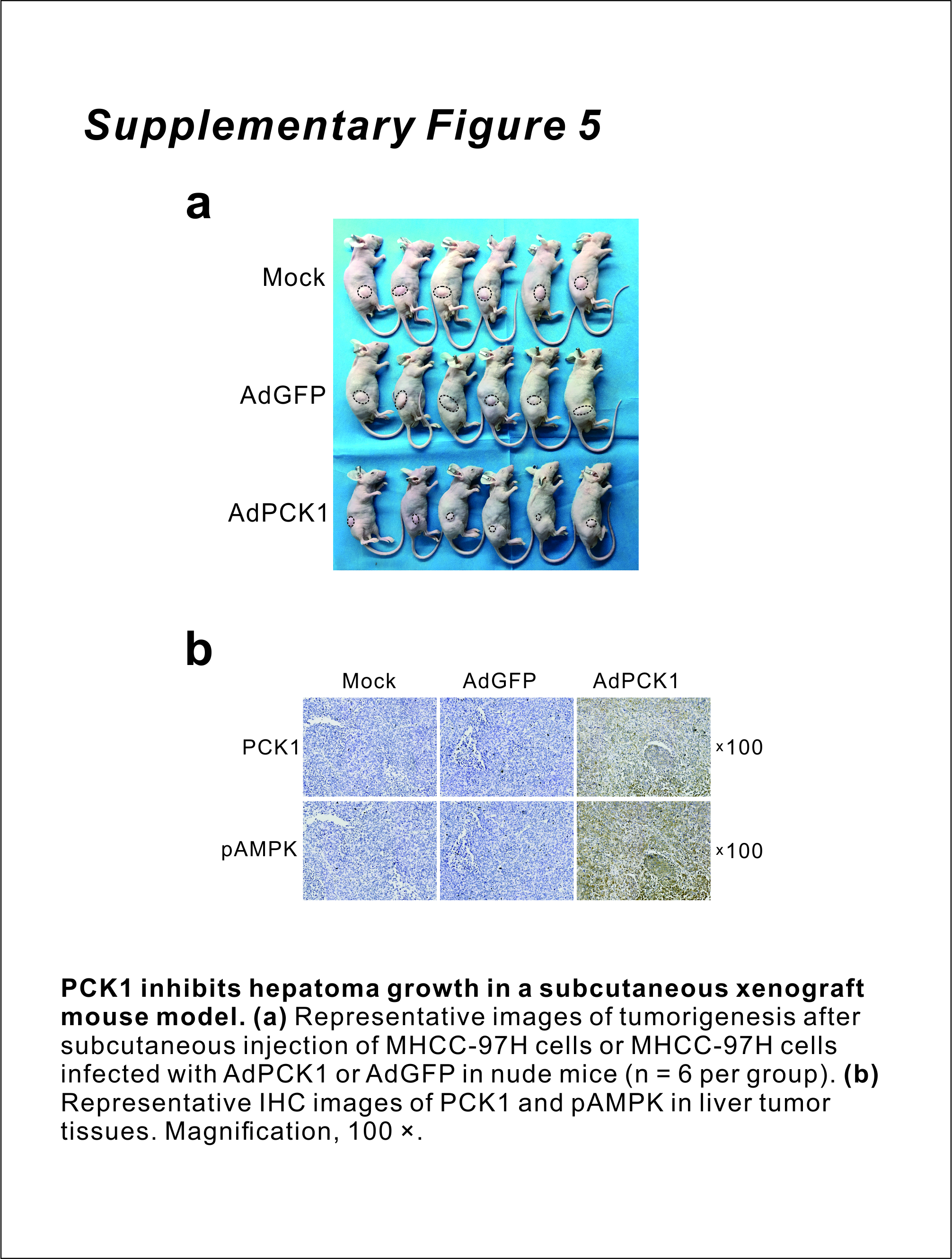

Supplement: Supplementary file 6 — PCK1 inhibits hepatoma growth in a subcutaneous xenograft mouse model. (JPG 3699 kb) [file 13046_2019_1029_MOESM6_ESM.jpg]
